# From Molecular Insight to Mesoscale Membrane Remodeling: Curvature Generation by Arginine‐Rich Cell‐Penetrating Peptides

**DOI:** 10.1002/smtd.202501539

**Published:** 2026-03-24

**Authors:** Katarína L. Baxová, Jovi Koikkara, Christoph Allolio

**Affiliations:** ^1^ Institute of Organic Chemistry and Biochemistry Czech Academy of Sciences Prague Czech Republic; ^2^ Faculty of Mathematics and Physics Charles University Prague Czech Republic; ^3^ Center for Advanced Systems Understanding Helmholtz Zentrum Dresden‐Rossendorf Görlitz Germany

**Keywords:** arginine magic, cell‐penetrating peptides, chemical specificity, molecular dynamics simulations, monte carlo simulations, multiscale modeling

## Abstract

The enhanced cell penetration ability of arginine‐rich peptides, such as nonaarginine ($R_{9}$), compared to their lysine‐rich counterparts, remains incompletely understood. Atomistic simulations reveal that $R_{9}$ binds significantly stronger (≈20kJ/mol) and penetrates deeper into the anionic lipid headgroup region than its lysine equivalent. This enhanced interaction translates into a stronger induction of negative membrane curvature by $R_{9}$. We introduce an integrative modeling workflow to extract and incorporate material properties from molecular simulations into a continuum membrane model that includes peptide binding and curvature induction. Our model predicts that stable membrane invaginations, as observed in studies of cell penetration, require excess membrane and are stable only for $R_{9}$. By analyzing lipid and protein sorting coupled to the membrane structure, we explain the interplay of Gaussian and mean curvature in providing a mechanistic basis for the initial membrane deformation events potentially involved in “Arginine Magic” cell entry pathways.

## Graphical abstract

Cell‐penetrating peptides are a widely‐used drug delivery platform. So far, there is a lack of quantitative understanding of the mechanisms that drive their efficiency. We introduce an integrative modeling approach combining molecular dynamics and continuum modeling, and apply it to show how the binding of cell‐penetrating peptides leads to membrane deformations, by a hydrophobic screening mechanism, which enables *Arginine Magic*.

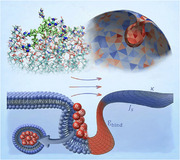

## 1 Introduction

Cell‐penetrating peptides (CPPs) are able to transport large, polar cargo across the plasma membrane, making them an integral part of many drug delivery platforms [1]. *Arginine Magic* denotes the particular ability of arginine‐rich CPPs to enter cells, even in conditions where endocytosis is impaired [2]. The positively‐charged peptide nonaarginine $R_{9}$ is an efficient CPP, in contrast to nonalysine ($K_{9}$), which has the same charge. This (molecular) ion‐specific effect has been studied on artificial phospholipid mixtures rich in phosphatidylethanolamine (PE) and phosphatidylserine (PS) as model systems for cell penetration [3, 4, 5]. It was found that these model membrane systems selectively undergo fusion and form multilamellar structures [3] as well as cubic phases upon $R_{9}$ addition [4, 5]. Analogous multilamellar structures have also been documented inside cells on multiple occasions [3, 6, 7].

Even though the actual cellular entry mechanism was reported to be dependent on heparin binding receptors [8], and membrane proteins were reported to be necessary for passive entry even into the giant plasma membrane vesicles [9], the structural analogies of the PE and PS model system have held up well ‐ even the experimentally known selectivity for arginine over lysine transfers to this model. Hence, model lipid bilayers have been extensively studied via molecular dynamics (MD) simulations [10, 11, 12, 13].

$R_{9}$ has often been reported to induce membrane curvature [3, 4, 14, 15, 16], either positive, negative, or negative Gaussian in nature. It is becoming a consensus that membrane curvature induction is a key component of the cellular entry mechanism [16]. Despite this, the precise mechanism by which these peptides induce membrane curvature is unknown. In the context of hydrophobic insertion and scaffolding, curvature generation by proteins is well‐understood [17, 18, 19, 20, 21, 22, 23, 24], but $R_{9}$ peptide is unstructured and hydrophilic. We recently established a framework for extracting membrane curvature elastic properties from molecular simulations [25, 26, 27]. Our method allows us to study the molecular basis of curvature generation. These results also extend to pore formation [28].

We revisit the molecular basis for*Argine Magic* in the domain of lipid binding, quantify and explain the induction of curvature in pure phospholipid bilayers and incorporate the resulting data into a model for arginine binding on mixed model bilayers, then integrate it into our recently developed general Monte Carlo (MC) toolkit for membrane deformations, which couples it to the mesoscopic structure [29].

This enables us to propose a new integrative modeling workflow, which allows us to investigate the conditions and physical determinants of inward budding as a proposed initial step of passive cell penetration. Most recently, Pei and coworkers have postulated a revised mechanism of entry for CPPs, which departs from inward budding, stabilized by *negative* membrane curvature [7].

## 2 Integrative Modeling

### 2.1 Workflow

**FIGURE 1:**
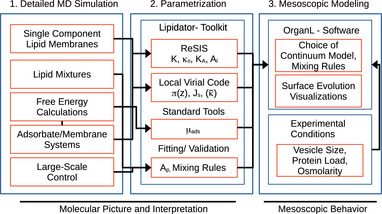
Schematic of Integrative Modeling Workflow.

MD simulations are a staple of research in chemistry, biology, and material sciences. Their chief limitations are their computational cost and lack of access to processes happening at very slow timescales. Our methodology allows to transfer the key effects of molecular interactions into a continuum model and evolve this model. To this effect, we have implemented an integrative modeling workflow, as outlined in Figure 1. This workflow can be readily generalized to arbitrary protein/membrane systems. It is particularly suitable to the interaction of fluid lipid membranes with unstructured proteins. In addition to the obvious advantage in simulation cost, it also has several less immediate benefits: 1. The parametrization procedure in the central panel of Figure 1 allows to map the effects of molecular composition on material properties and thereby provide a molecular interpretation of mesoscopic effects. 2. It allows to vary experimental conditions without rerunning detailed simulations. In particular, the effect of osmotic pressure is difficult to access with MD simulations. 3. As we have extensive tooling and documentation in place, and most parameters are derived directly from standard simulation techniques, our approach can be used by researchers without a strong background in traditional continuum methods. The general procedure is outlined in the following.

### 2.2 Continuum Model

The Helfrich–Canham–Evans theory has been a workhorse of mesoscale simulations of membrane systems for a long time. In contrast to the classical Helfrich [30, 31, 32] theory, the extended Helfrich–Kozlov–Hamm [33] (HKH) theory also includes lipid orientational information. It can be written as a surface free energy function:

(1) $$F=\int dA\{\frac{\kappa }{2}{\left(\tilde{H}-J_{S}\right)}^{2}+\frac{\kappa_{t}}{2}t^{2}+\bar{\kappa }\tilde{K_{G}}\}$$

In classical Helfrich theory, the free energy F is computed from a harmonic potential in the mean curvature H. The modified mean curvature $\tilde{H}$ contains two changes: First, in the HKH framework, it is computed from the covariant (surface) divergence of the membrane director vectors ∇·n instead of the usual surface normals. Second, the usual factor of $\frac{1}{2}$ is omitted, so that for lipid directors aligned with the surface normals $\tilde{H}$ is the sum of principal curvatures. The parameters κ and $J_{s}$ refer to the bending rigidity and the membrane's intrinsic curvature. The energetic cost for tilting a lipid out of the membrane plane with a vector t is associated with the tilt modulus $\kappa_{t}$. Finally, there is a contribution from the Gaussian curvature $K_{G}$, with bending modulus $\bar{\kappa }$. The tilt degree of freedom was originally introduced to construct a model of membrane fusion [34].

### 2.3 Parameter Extraction from Molecular Dynamics

In a fluid membrane, the tilt has a short correlation length. However, its fluctuations give access to κ, as tilt contributes to director divergence. This relation can be exploited either using Fourier‐space methods [35] or real‐space fluctuations. We use an instantaneous membrane surface interpolation [36] and atom‐set based descriptor definitions to directly compute ∇·n, the ReSIS real‐space method. The separation t≈n−N [37] allows us to compute $\kappa_{t}$ and, in particular, κ from an equilibrium probability distribution:

(2) $$P\left(\nabla \cdot t\right)\propto \exp\left[-\frac{1}{2}\kappa \beta {\left(\nabla \cdot t\right)}^{2}A_{l}\right]$$

Here, $A_{l}$ represents the area per lipid, which is also a simulation output. The Gaussian probability distribution is the result of the harmonic potential of the tilt divergence.

The lateral stress profile π(z) has long been established to give access to the membrane properties  [38, 39, 40, 41]. In order to compute it, we first need to obtain the local stress tensor σ(x). It is calculated as a sum of the kinetic part $\sigma_{K}$, and the potential part $\sigma_{V}$. $\sigma_{K}$ is trivially computed from the velocities, but the potential‐dependent part contains an ambiguity [42]:

(3) $$\sigma_{V}{\left(x\right)}^{\alpha \beta }=-\frac{1}{2}<\sum_{i\neq j}f_{\mathrm{ij}}^{\alpha }\int_{C_{i,j}}\delta \left(x-l\right)ds^{\beta }>$$

The contour integral in the above expression can, in principle, be chosen freely (here the line element is **s** and its position vector **l**). In addition, the two‐body force $f_{ij}$ cannot be uniquely determined for many‐body potentials. We follow a pragmatic approach, choosing the Goetz–Lipowsky [43] decomposition (GLD) for numerical stability and the Harasima [44] contour (following cartesian components) for the possibility to use Fourier‐space methods for the Ewald summation. The corresponding code was implemented by Sega et al. [45] and supplemented with the GLD by us. The lateral pressure profile π(q) is then computed by subtracting the normal components $\sigma {\left(q\right)}_{⊥}$ from the lateral ones $\sigma {\left(q\right)}_{∥}$ along some axis of symmetry, parametrized by q.

(4) $$\pi \left(q\right)=\sigma {\left(q\right)}_{∥}-\sigma {\left(q\right)}_{⊥}$$

The choice of the Harasima contour requires manual setting of the normal pressure $p_{N}$, which is required to be constant by mechanical stability. As we know our membranes to be tension‐free, we obtain $p_{N}$ from.

(5) $$\int dz[\pi \left(z\right)-p_{N}]=0$$

Here, the integration is carried out along the membrane normal ${\hat{e}}_{z}$. By combining the computed stress profiles with the elastic properties, we can now use the established relationship [33, 38] between the first bending moment and the product of spontaneous curvature, $J_{s}$ and κ,

(6) $$\kappa J_{s}=\int_{0}^{l}\pi \left(z\right)zdz$$

to compute $J_{s}$. This procedure enables a local computation, due to the locality of the ReSIS κ. In order to extract parameters within our workflow (see Figure 1), parameters need to be extracted from simulations of single‐component lipid membranes as well as membrane‐adsorbate systems.

### 2.4 Lipid Mixing

The traditional approach is to calculate the properties of lipid compositions using the area fraction $\phi_{i}$ of individual lipids [46, 47]. Accordingly, the local spontaneous curvature $J_{s}$ is computed as:

(7) $$J_{s}=\sum_{i}\phi_{i}c_{i}^{0}$$

where $c_{i}^{0}$ is the spontaneous curvature of the individual lipids. Concerning bending rigidity, a harmonic average is used:

(8) $$\frac{1}{\kappa }=\sum_{i}\phi_{i}\frac{1}{\kappa_{i}}$$

We recently validated these classical approaches using a direct comparison with complex membranes and their properties [25].

Another (traditional) assumption we make is ideal mixing. Ideal mixing generates large entropies, which usually prevents what we call lipid demixing. By lipid demixing, we mean that a large excess or depletion of specific lipids occurs in a region of the membrane. Traditionally, this phenomenon is associated with domain formation and phase separation [48, 49]. Obviously, in such a situation ideal mixing does not apply. Domain formation, usually driven by sterols, leads to lipid demixing and the emergence of line tension. A very large body of literature has discussed these effects in the context of protein‐membrane interactions, including protein‐driven formation of lipid rafts, which might exhibit line tension [50, 51, 52, 53].

In a purely unsaturated phospholipid system, no such effects can be expected (though our code supports line tension on domain boundaries). Any lipid demixing will strictly be the result of peptide binding and peptide coverage in conjunction with curvature sorting. Lipid composition is modified in our code using a lipid swapping move, which moves two specific (random) lipids by a random (noninteger) amount between faces, while conserving the total reference area. The algorithm first selects an edge at random and then moves lipids across with a step size from a uniform distribution in a random direction (controlled by the sign). Monolayer parameters of both leaflets are then added up for bilayer values (see the Supporting Information).

### 2.5 Adsorbate and Counterion Effects

The effect of counterions and electrical charge on membrane properties has been extensively studied, mostly within classical Poisson–Boltzmann theory [54, 55, 56, 57, 58]. We circumvent the issues of ion and molecular specificity, e.g. for $R_{9}$, by simply computing the values of the parameters ($\kappa ,J_{S},\text{…}$), with the same methods used for lipids. We then combine it with the mixing rules using an adsorbate coverage fraction,

(9) $$\phi_{p}=n_{p}\frac{A_{p}}{A_{l}}$$

where $n_{p}$ is the number of adsorbed peptides. $\phi_{p}$ is controlled not to exceed 1. $A_{l}$ is the total reference area of the lipid patch. Note that this approach requires setting a protein area $A_{p}$. For example, the resulting intrinsic curvature is generated as:

(10) $$J_{s}=\phi_{p}\left(\sum_{i}J_{s,i}^{p}\right)+\left(1-\phi_{p}\right)J_{s},n$$

where the subscript $J_{s,i}^{p}$ is spontaneous curvature of the lipid‐adsorbate system for lipid component i and $J_{s},n$ is the free lipid mixing result from Equation (7). See the SI for the full details.

In case of a strong preferential binding of adsorbates to specific lipids, ideal mixing can no longer be assumed. Free energy calculations allow us to compute the binding energy $\mu_{ads}$ to single component lipids and mixtures from MD simulations. One possible way to extrapolate these data to lipid mixtures is to assume that the free energy of binding $F_{b}$ can be computed from the lipid fractions as.

(11) $$F_{b}=n_{p}\sum_{i}\phi_{i}\mu_{ads,i}$$

This assumption needs to be validated for each adsorbate. We have also implemented and tested the possibility of adding arbitrary interaction potentials between surfaces to our code (see the accompanying manual) so that electrostatic effects can, in principle, be added. We have refrained from doing so in this case, as we believe that the net charge of the membrane is small, where (adsorbate bound) surfaces are in close contact. We are still lacking a good theory to parametrize, in place of the Poisson–Boltzmann framework, which does not appear suitable due to the high charge of individual ions, the “ion specificity” of amino acids, and elevated membrane surface charge. Adsorbates are moved along the membrane like lipids, except that only integer populations are allowed.

### 2.6 Gaussian Curvature

In a closed surface, the Gaussian curvature integral is a topological invariant (Gauss–Bonnet theorem). However, where lipid composition symmetry is heterogenous, the resulting local changes in $\bar{\kappa }$ lead to a nontrivial contribution of Gaussian bending. The Gaussian bending modulus of a single lipid monolayer is notoriously difficult to determine, as it is only accessible from open membranes or during topological changes [59]. In principle, it is available from the second moment of the lateral stress distribution. However, the determining $\bar{\kappa }$ in this way requires an exact knowledge of the pivotal plane of the membrane and is unreliable [59]. An alternative is to compute the bilayer Gaussian bending modulus by integrating over both leaflets.

(12) $${\bar{\kappa }}_{b}=\int_{-l}^{l}\pi \left(z\right)z^{2}dz$$

We have previously reported $\bar{\kappa_{b}}$ obtained in this way [27], and have added pertinent computed values to the Supporting Information. Nevertheless, we follow a different route. Numerical investigations [59] and previous experimental results [60] confirm that the monolayer Gaussian bending modulus is ca. 0.8–0.85 ·−κ, even for those lipids which are known to be susceptible to form cubic (nonzero $K_{G}$) phases. Expanding around the bilayer midpoint to linear order in the membrane thickness d gives the following relation [60]:

(13) $${\bar{\kappa }}_{b}=2\bar{\kappa }-4J_{s}\kappa d$$

which is what we use together with our estimate of $\bar{\kappa }$. While this adds more realism, in practice, the contribution of Gaussian bending to membrane stability is not large when using this approximation.

### 2.7 Surface Evolution

Classical Dynamically Triangulated Surface (DTS) methods [61, 62] are based on operator discretizations on either vertices or edges. We have recently developed an alternative approach, OrganL [29], in which edge interpolants are assembled into *quasi*‐tangent continuous curved faces. This approach, in principle, enables the evaluation of the full shape operator (including off‐diagonal elements) and continuous functions on membrane patches, without sacrificing the ease of parallelization and locality offered by DTS. Our method is based on an idea by Nagata [63], who developed a quadratic edge interpolant. The interpolant, φ(t), is identical to the one proposed by Nagata, wherever the latter gives reasonable results.

(14) $$\vec{\varphi }\left(t\right)=x_{A}+\left(d-c_{1}\right)t+c_{1}t^{2}\left[-c_{2}t+c_{2}t^{3}\right]$$

The Nagata interpolant imposes an orthogonality condition of the tangent vector t at vertex points ($x_{A,B}$) to the vertex normals n,

(15) n·t=0

This leads to an underdetermined system for the coefficienct c, which is solved by a Moore–Penrose inverse, resulting in an analytical construction. It was soon discovered that this interpolant fails for a wide variety of normal orientations [64]. We developed an extension by increasing the interpolation order (square brackets of Equation (14)), and additionally requiring orthogonality to an estimate of the binormal vector, based on the vector d connecting the vertices,

(16) (d×n)·t=0

To provide a closer approximation to tangent continuity, a penalty is added (see ref. [29]). The edge interpolants are assembled into patch interpolants on triangles and the structure is evolved with a Metropolis Monte Carlo algorithm, as is common for DTS simulations, generating a canonical distribution with the normal vectors as additional degrees of freedom.

## 3 Application to Cell‐Penetrating Peptides

### 3.1 Peptide Binding to Pure and Mixed Lipid Bilayers

#### 3.1.1 Pure Membranes

**FIGURE 2:**
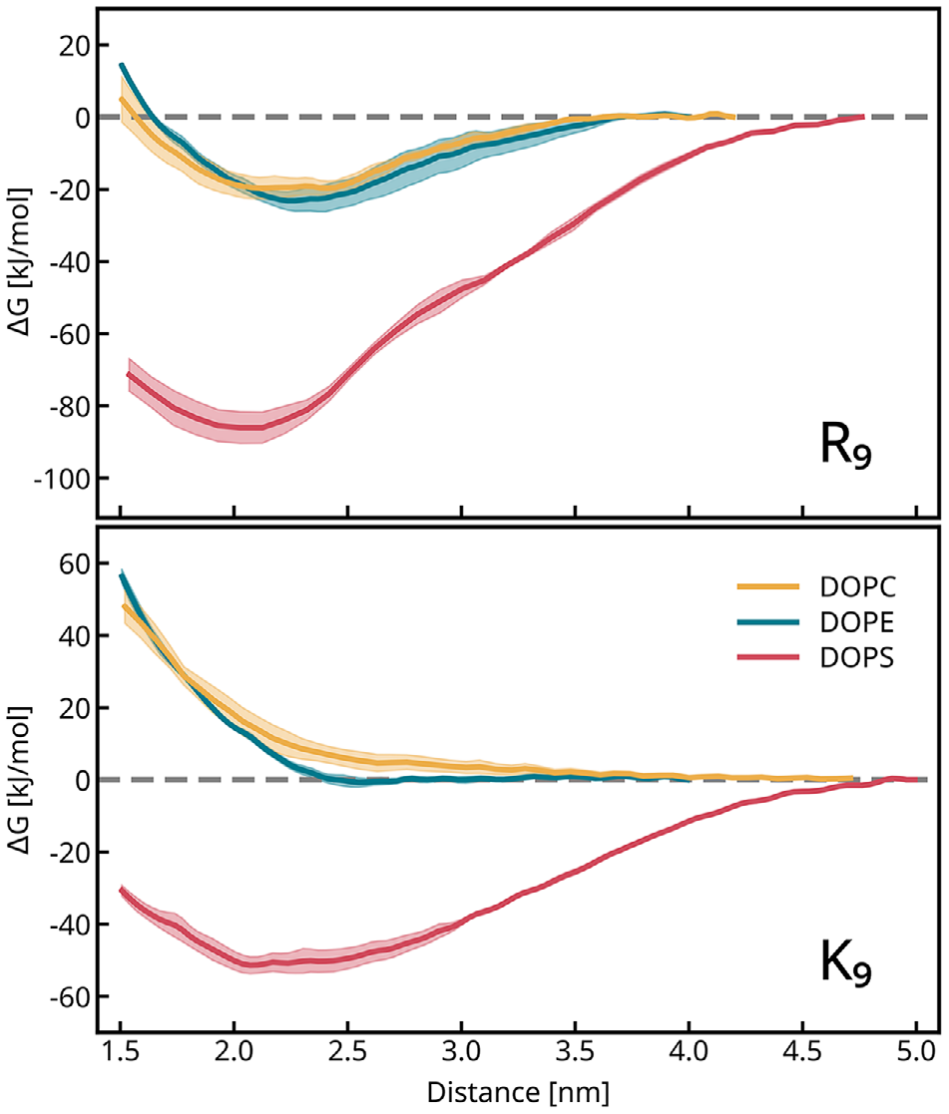
Top panel: Free energy profile of a single $R_{9}$ binding to a lipid patch composed of DOPC (yellow), DOPE (teal), and DOPS (red). Bottom panel: equivalent energy profiles for membranes interacting with $K_{9}$. The dashed grey line emphasizes zero free energy.

We studied membrane adsorption and lipid selectivity of the CPPs by computing the Potentials of Mean Force (PMF) of peptide binding to single‐component lipid bilayers. PMFs are the free energies along a chosen coordinate, in this case the center of mass distance between membrane and peptide along the membrane normal (z‐coordinate). The PMFs (Figure 2) consistently show stronger binding of $R_{9}$ over $K_{9}$ to lipid membranes, in particular to the pure DOPS bilayer, where the difference is >20kJ/mol (see Table 1) for the binding energy values and errors. We find no significant binding of $K_{9}$ to the neutral PC and PE bilayers, however, the repulsion is lower for DOPE. There also appears to be a slightly higher binding affinity to DOPE for $R_{9}$. The difference between the charged DOPS and the other lipids is mainly due to the electrostatics of binding. We used the scaled‐charge ProsECCo forcefield for the simulations to obtain binding free energies [65]. When comparing with published work [66], we estimate that our binding free energies are slightly lower than when using an unscaled force field. The binding energy difference between $K_{9}$ and $R_{9}$ with PS is larger than for the other bilayers, hinting at a specific interaction.

**TABLE 1:** Binding Energy for protein‐lipid bilayer system Binding free energies ($\Delta G_{\text{bind}}$) of two peptides ($R_{9}$ and $K_{9}$) to different lipid bilayer compositions, obtained from molecular dynamics simulations. The values are reported in kJ/mol with associated standard error.

$\Delta G_{\text{bind}}\;\text{[kJ}/\text{mol]}$	$R_{9}$	$K_{9}$
DOPE	−23.2 ± 2.6	−0.7 ± 1.3
DOPS	−86.0 ± 4.3	−51.4 ± 2.3
DOPC	−19.6 ± 3.0	×
Mixture	−50.0±1.4	−23.3±0.8

Yet, the long‐range electrostatics do not differ (due to the identical sidechain charge). This implies that if the binding energy is part of the “magic”, it must lie in the stronger binding of arginine sidechains to membranes.

#### 3.1.2 Mixed Membranes

We report binding energies of the peptides to mixed membranes in Table 1. Peptide binding to the mixtures is stronger than to pure neutral lipids but weaker than to the charged pure DOPS.

Next, we present results from our large‐scale mixed‐membrane simulation. The membrane contains all examined lipids at the ratios DOPE:DOPS:DOPC 60:20:20. Analyzing this simulation enables us to disentangle the molecular picture at the interface.

**FIGURE 3:**
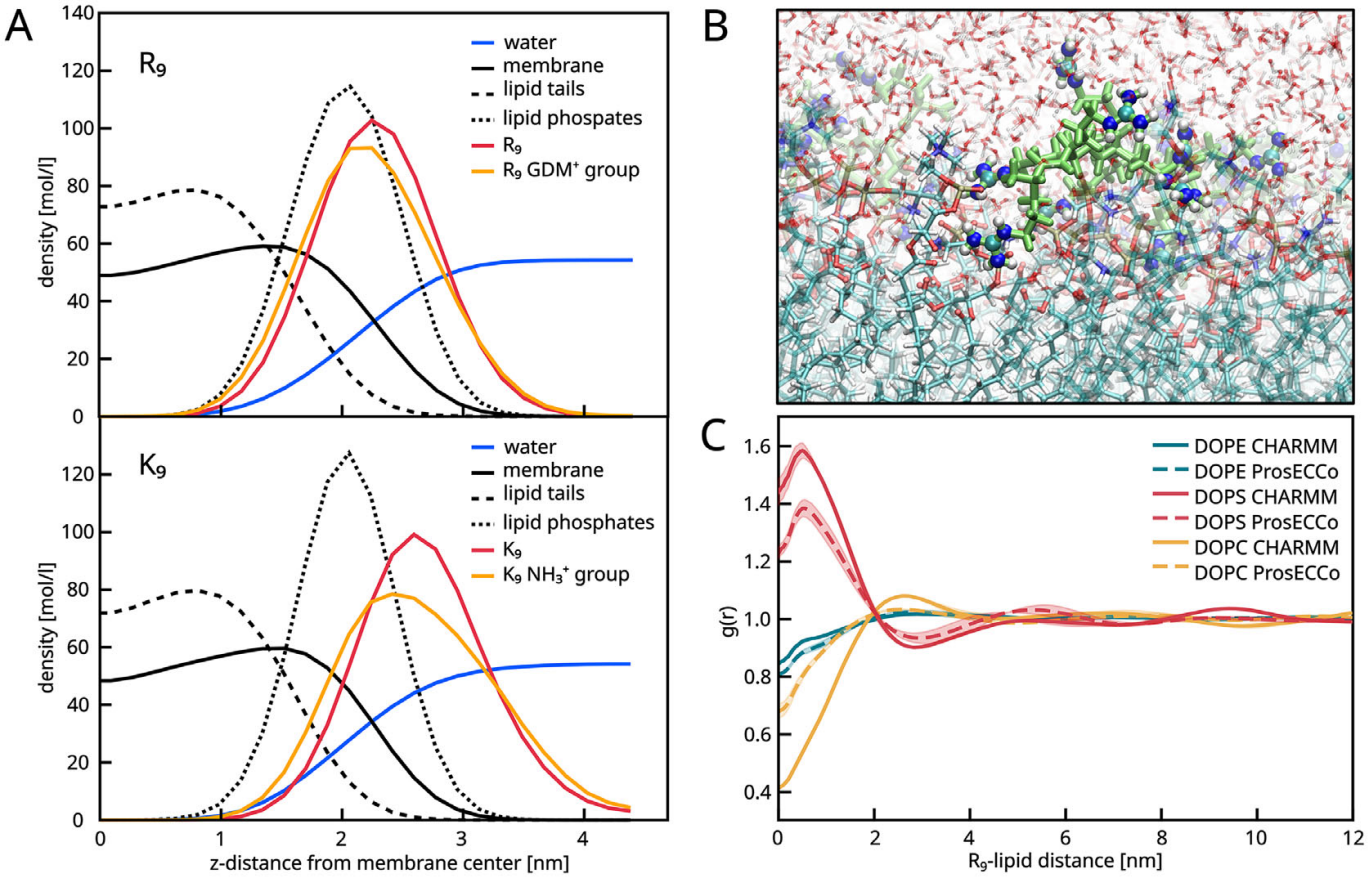
A: Density profiles from the large‐scale simulations of $R_{9}$ (top) and $K_{9}$ (bottom) centered on the membrane midplane, showing CHARMM results only. Density profiles of all membrane atoms (black, solid), water (blue), lipid tail C‐atoms (black, dashed), phosphate P atoms (black, dotted), peptides (red), and peptide headgroups (orange). B: Simulation snapshot showing nonaarginine (green, with emphasized guanidinium parts) at the membrane surface. Water is rendered in red and white, membrane lipid tails in cyan. C: 2D‐RDFs of lipids around $R_{9}$: $R_{9}$‐DOPE RDF in teal (solid and dashed for CHARMM and (scaled) ProsECCo, respectively); $R_{9}$‐DOPS RDF in red and $R_{9}$‐DOPC RDF in yellow.

In Figure 3A, we compare the density profiles of different species at the membrane interface in the presence of the peptides. The striking difference between $R_{9}$ and $K_{9}$ lies in the deeper binding of the arginine sidechains inside the membrane (orange, upper part) compared to the lysine sidechains (orange, lower part). The arginine sidechain density is collocated with the headgroup/phosphate position, whereas lysine is mainly distributed above the headgroup area. The deeper penetration of the arginine sidechains brings it closer to the locus of negative charge. This enhances the electrostatic interaction energy as well as the mutual screening of the charges. The picture is similar to the hydrophobic insertion mechanism by Kozlov et al. [22], except that in this case, the interaction is ionic and not hydrophobic, and the steric component is minor. Yet, it is widely known that arginine is more hydrophobic than lysine, and we believe this is part of the explanation for why arginine is able to enter membranes more deeply.

#### 3.1.3 Arginine‐Arginine Interaction

Past studies showed that in solution, guanidinium moieties of the arginine sidechains do not repel, but interact weakly [10]. Aggregation of $R_{9}$ on membranes has been observed in simulation [12]. Additionally, R9 aggregation in water has been recently observed in experiment [67]. In contrast, the binding of $R_{9}$ to membranes charged at 20% was previously found not to be cooperative [11]. [Correction added on March 31, 2026, after first online publication: citations have been updated.]

We computed 2D radial distribution functions (RDF)s of a large number of $R_{9}$ molecules on a flat lipid patch (see Methods). The results are shown in Figure S1. These data are not consistent with a strong binding between poly‐$R_{9}$ molecules, as the RDF does not exhibit major peaks, but fluctuates around unity away from a depletion/exclusion zone, in agreement with the data by Robison et al. [11]. Therefore, we suggest treating $R_{9}$ chains as noninteracting on (negatively charged) lipid membranes.

#### 3.1.4 Arginine‐Lipid Demixing

We have already found that $R_{9}$ strongly interacts with lipids, particularly with DOPS. The long‐term large‐scale MD simulations of the membrane with peptides (snapshot in Figure 3B) allow us to compute realistic 2D RDFs of the lipid molecular centers of mass vs. $R_{9}$ at the membrane surface (Figure 3C). The strong binding of $R_{9}$ to negatively charged PS lipids is enough to generate a significant amount of demixing This is evidenced by a high peak of PS under the protein, accompanied by depletion of the other lipids. This depletion is larger for DOPC than for DOPE, as observed in both charge‐scaled and unscaled simulations. It suggests a strong $R_{9}$ preference toward PS at the expense of PC local lipid density, while PE remains almost unaffected. We report estimates on an excess number of lipids per peptide (Kirkwood–Buff integrals) and the peak of RDF in Table 2.

**TABLE 2:** Characteristic quantifiers of protein‐lipid preferential interactions. Γ and $g_{\text{ext}}$ are the excess number of lipids per peptide and the extremum of the first RDF peak in Figure 3C, respectively. Errors in Γ and $g_{\text{ext}}$ are the absolute differences between the mean (averaged over “ProsECCo” and “CHARMM”) and individual model values.

$R_{9}$	Γ	$g_{\text{ext}}\left(r\right)$
DOPE	0.3 ± 0.6	0.8 ± 0.02
DOPS	1.3 ± 0.7	1.48 ± 0.10
DOPC	−1.7 ± 1.4	0.54 ±0.13

A high peptide concentration was chosen to improve sampling. However, it also means that of the 32 lipids for each protein, a significant amount will be in direct contact. This naturally limits the amount of lipid demixing, so the amount of lipid selectivity is likely not as high as at lower concentrations.

An earlier study by Khelashvili et al. using a Poisson–Boltzmann/ideal mixing‐based [68] found only weak demixing of PS in the proximity of a single adsorbed $K_{13}$ molecule. The highest increase in PS was a factor of ≈1.5, which is not very different from our observed RDF peaks.

### 3.2 Peptide Binding Effect on Material Properties

We computed the bending moduli κ and a bilayer tilt moduli $\kappa_{\Theta }^{b}$ with the ReSIS method, based on local tilt fluctuations [36, 69]. We previously computed these values for DOPE, DOPC, and DOPS lipid membranes, but recomputed the value for DOPS to control the reproducibility of previously published simulation results [27]. To examine the influence on membrane bilayer properties, we put the membrane in contact with high (charge‐neutralizing) loads of $R_{9}$, which we estimate to be close to saturation.

#### 3.2.1 Material Properties

To understand how $R_{9}$ and $K_{9}$ modify the material properties of lipid bilayers, we performed simulations on single‐component bilayers. We used 6 $R_{9}$ for uncharged lipids and 14 peptides for charged membranes to achieve approximate charge neutrality. The results, including equilibrium areas per lipid ($A_{0}$), are in Table 3. Since $K_{9}$ does not bind to the uncharged lipids, as per our PMFs, we did not simulate the corresponding membranes. The influence of the peptides on elastic properties is not large for either peptide. However, it appears that $K_{9}$ stiffens DOPS membranes, while $R_{9}$ might slightly soften them. This effect is also visible in the area per lipid and might be attributed to a surface tension contribution of $K_{9}$, which is offset by increased chain‐packing. In contrast, the entry of $R_{9}$ into the headgroup region might counteract some of the compressive effects of charge screening.

**TABLE 3:** Simulation results for bilayer mechanical properties. Bending moduli κ and spontaneous curvatures $J_{s}$ are monolayer quantities. Areas per lipid $A_{0}$ and tilt moduli $\kappa_{\Theta }^{b}$ are bilayer properties. Errors are standard errors.

System	κ [kT]	$J_{s}[{\mathrm{nm}}^{-1}$]	$A_{0}$ [Å 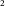 ]	$\kappa_{\theta }^{b}\left[kT/{\mathrm{nm}}^{2}\right]$
${\mathrm{DOPE}}^{a}$	15.83	−0.24	61.64 ± 0.16	14.76
${\mathrm{DOPC}}^{a}$	11.56	0.0	68.02 ± 0.08	22.68
DOPS	14.27 ± 0.84	−0.06 ± 0.01	63.38 ± 0.23	19.76 ± 1.23
DOPE + 6 $R_{9}$	16.20 ± 0.80	−0.30 ± 0.03	61.09 ± 0.14	23.15 ± 0.94
DOPC + 6 $R_{9}$	10.88 ± 0.49	−0.03 ± 0.02	68.60 ± 0.17	14.31 ± 0.56
DOPS + 6 $R_{9}$	14.37 ± 0.61	−0.13 ± 0.03	62.48 ± 0.26	21.96 ± 0.94
DOPS + 14 $R_{9}$	13.16 ± 0.60	−0.26 ± 0.08	63.23 ± 0.14	19.48 ± 0.96
DOPE + 6 $K_{9}$	not binding acc. to PMF			
DOPC + 6 $K_{9}$	not binding acc. to PMF			
DOPS + 14 $K_{9}$	16.26 ± 0.52	−0.17 ± 0.02	61.49 ± 0.40	23.71 ± 0.84

*Source*: From [25] unscaled simulations at 303.15K.

#### 3.2.2 Stress Profiles

**FIGURE 4:**
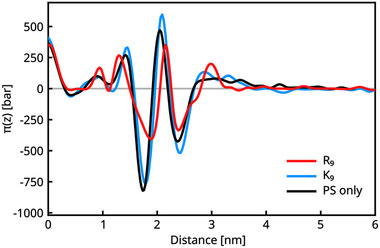
Comparison of the symmetrized lateral stress profiles of DOPS‐containing membrane in the presence of $R_{9}$ (red), $K_{9}$ (blue), and in the absence of peptides (black). On the x‐axis, z denotes the distance from the membrane center, projected on the surface normal. The grey line highlights 0 lateral stress value.

We computed local stress profiles for single‐component lipid membranes. The largest impact on the stress profile is found for the pure DOPS membrane. Figure 4 illustrates the different effects of $R_{9}$ and $K_{9}$ on charged lipids. We interpret the peak around 2nm as associated with headgroup repulsion. It is lower for $R_{9}$ than for $K_{9}$. The whole profile is damped for $R_{9}$, potentially also due to packing effects, but the decay of the positive electrostatic pressure in particular is faster for $R_{9}$. The positive pressure at large z is due to the electrostatic repulsion of the adsorbed proteins. We attribute the lower repulsion found for $R_{9}$ both to the electrostatic screening by headgroup binding and, to some extent, to the compactness of the peptides due to the attraction between sidechains.

#### 3.2.3 Curvature Generation

A large negative curvature generating effect is observed for $R_{9}$ on DOPS (see $J_{s}$ in Table 3). This result is similar to what was observed in the case of ${\mathrm{Ca}}^{2+}$ ions [27] on the same lipid. In our previous study [27], ${\mathrm{Ca}}^{2+}$ was shown to stabilize DOPS membrane fusion stalks as indicative of fusion. Note also that ${\mathrm{Ca}}^{2+}$ experimental behavior was similar to that of CPPs in this system [3]. The effect of $R_{9}$ on the bending moment $\kappa J_{s}$, see Equation (6), is significantly stronger than that at the same loading of $K_{9}$. Furthermore, the increased κ on $K_{9}$ reduces the effect on $J_{s}$. We find that $R_{9}$ can even reduce the spontaneous curvature of uncharged DOPE. Previously, we found pure DOPE/DOPS to be very susceptible to fusion by $R_{9}$ (over $K_{9}$); fusion activity was reduced by DOPC [3].

The error bar values in the curvature calculations (Table 3) are standard errors, including error propagation (see Supporting Information). The $J_{s}$ values given here were calculated by integration over the whole box using Equation (6). We also computed the Gaussian bending rigidity $\bar{\kappa }$ for selected systems, from the second moment of the lateral stress distribution from Equation (12). Finding a stabilization of the Gaussian curvature by $R_{9}$ and, to a lesser extent of $K_{9}$. These results are given in Table S1, but are not used further, as we have low confidence in this way to obtain $\bar{\kappa }$.

### 3.3 Choice of Continuum Model

The basis of our model is the Helfrich–Canham–Evans theory [30, 31, 32]. We do not use the full HKH theory, as we do not expect the membrane tilt to play a major role. Instead of constraints for area and volume, we introduce laterally compressible membranes and an osmotic pressure [70], as well as regular solution type mixing terms. The total energy of the system is then given by

(17) $$\begin{array}{cc} F=E_{\text{HF}}+E_{\text{pV}}+ & E_{\text{stretch}}+\sum_{\text{face}}\left[F_{b}+F_{\text{mix, b}}\right] \end{array}$$

where

$$\begin{array}{cc} E_{\text{HF}} & =\int_{\text{Surf}}dA\left[\frac{\kappa }{2}{\left(2H-J_{S}\right)}^{2}+{\bar{\kappa }}_{b}K_{G}\right]\\ E_{\text{pV}} & =c_{0}k_{B}T\left[\left(V-V_{0}\right)-V_{0}\log\left(V/V_{0}\right)\right]\\ E_{\text{stretch}} & =\frac{K_{A}}{2}{\left(1-\frac{A}{A_{0}}\right)}^{2}\\ F_{b} & =n_{p}\sum_{i}\phi_{i}\mu_{ads,i}\\ F_{\text{mix, b}} & =k_{B}TM\sum_{i,b}\phi_{i}\ln\phi_{i} \end{array}$$

Here H denotes the mean curvature (in the sense of $\tilde{H}$ without tilt), the parameters for (Gaussian) bending rigidities ($\bar{\kappa }$) κ and spontaneous curvature $J_{S}$ vary over the mesh but are constant on each face. Integration is performed on the faces. The bilayer binding energy of the peptide $F_{b}$ and the mixing free energy $F_{\text{mix, b}}$ are calculated per face and added for a total lipid population M. The volume work $E_{\text{pV}}$ is calculated using the vesicle volume V from work against the osmotic pressure difference between the interior and exterior of the vesicle (assuming balanced osmolarity at $V_{0}$) at a solution osmolarity $c_{0}$. The area compression energy $E_{\text{stretch}}$ is calculated from the total area A using the bulk modulus $K_{A}$ and the stress‐free reference area $A_{0}$. $n_{p}$, $\phi_{i}$, and $\mu_{ads,i}$ are the local (mesh element) numbers of peptides, peptide coverage, and binding energy, respectively. See the Supporting Information for exact parameter values. Proteins modify the properties only of those lipids that they “cover” (discussed below and in Section 3.1.3). In our discretization, a protein will always cover exactly one triangle, there are no fractional occupations. For the binding free energies $\mu_{ads,i}$ of the lipids to the peptides, we utilise values within the error bars of our single‐component PMF data. All model parameters are summarized in Table S3.

#### 3.3.1 Model Validation

The only unfixed parameter of the model is the coverage area of $R_{9}$ on the membrane ($A_{p}$), which also determines the mesh resolution, as we only admit full coverage of a triangle. We optimized the area of $R_{9}$ to reproduce the lipid demixing of the large membrane system, as measured by the preferential interactions (see the Supporting Information). The optimal value was $14.786\;{\mathrm{nm}}^{2}$, resulting in the excess number of lipids per peptide, Γ, as:

(18) $$\begin{array}{cc} & \Gamma (DOPC)=-1.772,\;\Gamma (DOPS)=1.328,\\ & \Gamma (DOPE)=0.444 \end{array}$$

These results are in a good agreement with Table 1. To give an idea of the molecular dimension, this area corresponds to a radius of ca. 2.2nm, not too far from the RDF data, and providing a satisfactory resolution.

To further validate this approach, we considered a small system of 64 lipids and only one peptide. In this case, the coverage ratio is only 32.8%. For such a system, we predict a binding energy of −56.69kJ/mol, in reasonable agreement with the preferential interaction data in Table 1. Please note that demixing is predicted to be stronger in this system than in the large‐scale simulation with high peptide concentration, which leads to stronger binding. For $K_{9}$, we use the respective $\mu_{ads,i}$ ($=\Delta G_{bind}$) from Table 1 and the identical area as for $R_{9}$. Then the $K_{9}$ binding energy prediction for the small patch is −27.7kJ/mol, which is in good agreement with Table 1. Note that as $K_{9}$ has a lower binding energy and has experimentally anti‐cooperative behavior [11], the model for $K_{9}$ should be considered more of an upper bound of the effect, at least at identical peptide load. Our model reproduces preferential binding, binding free energies on lipid mixtures, and the demixing under peptides. In addition, when comparing partial and full load of $R_{9}$ on DOPS, we see an approximately linear increase, which supports our mixing scheme in presence of peptides.

### 3.4 Mesoscopic Consequences of $R_{9}$ Specificity

#### 3.4.1 Stability and Morphology

**FIGURE 5:**
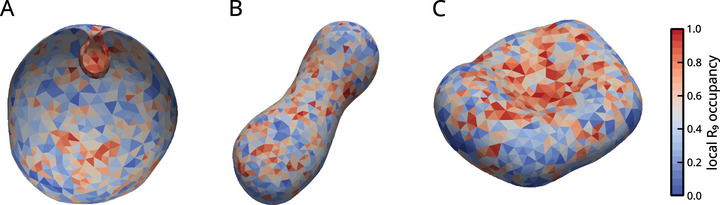
Representative structures with thermally averaged $R_{9}$ occupancy. A: Transverse cross section of stomatocyte of $\nu_{0}=1.00$, B: prolate of $\nu_{0}=0.75$ and C: discocyte of $\nu_{0}=0.80$.

The equilibrium shapes of vesicles governed by the Helfrich energy with uniform $J_{s}$ have been extensively studied [29, 71, 72]. Stable shapes include stomatocytes, discocytes, as well as prolate and oblate spheroids. In Figure 5, we display the representative morphologies captured during our simulation runs. It is important to note that they do not necessarily represent the long‐term stable equilibrium states. Helfrich minima are usually plotted as a function of the reduced volume:

(19) $$\nu_{0}:=\frac{6V_{0}}{{\left(A_{0}\right)}^{3/2}}\sqrt{\pi }$$

In a $J_{s}=0$, constraint optimization of the Helfrich functional $\nu_{0}$ is sufficient to characterize the geometry that minimizes the energy, due to a scale invariance of the system. In this setting, volume and area constraints are unified into one parameter. In our case, the values of $\nu_{0}$ correspond to the osmotic pressure difference minima of the free energy (see Equation (17)), i.e. zero resultant osmotic pressure ($V_{0}$) and the stress‐free area ($A_{0}$). While $\nu_{0}$ reflects the uncompressed area and volume, the instantaneous shape of the vesicle may vary due to thermal noise and mechanical forces. The stable shapes we obtain are also not scale invariant, so the use of $\nu_{0}$ serves mainly as a way to compare results with the best‐known system. As $\nu_{0}$ is linear in the volume, it can be interpreted as a degree of *filling* of the vesicle. Due to fluctuations, the geometry at $\nu_{0}=1$ is only approximately spherical.

Without spontaneous curvature, prolate configurations remain stable up to $\nu_{0}\approx 0.65$ [71], while stomatocytes are global free energy minima for $\nu_{0}$ lower than ≈0.55. Between these two values, the discocyte structure is stable. Our model system is not scale‐invariant, as the individual lipids have defined intrinsic curvatures, and we use an extended energy functional. Hence, the results are valid only for our choice of vesicle size and osmolarity. The choice of osmolarity and vesicle sizes reflects the conditions of previously performed experiments [3]. Here, we investigate the stabilization of competing structures in the presence of lipids and peptides, with a focus on the “stomatocyte” shape. This geometry is of primary interest as it represents inward budding, a process hypothesized to be the initial step of CPP entry [7].

**FIGURE 6:**
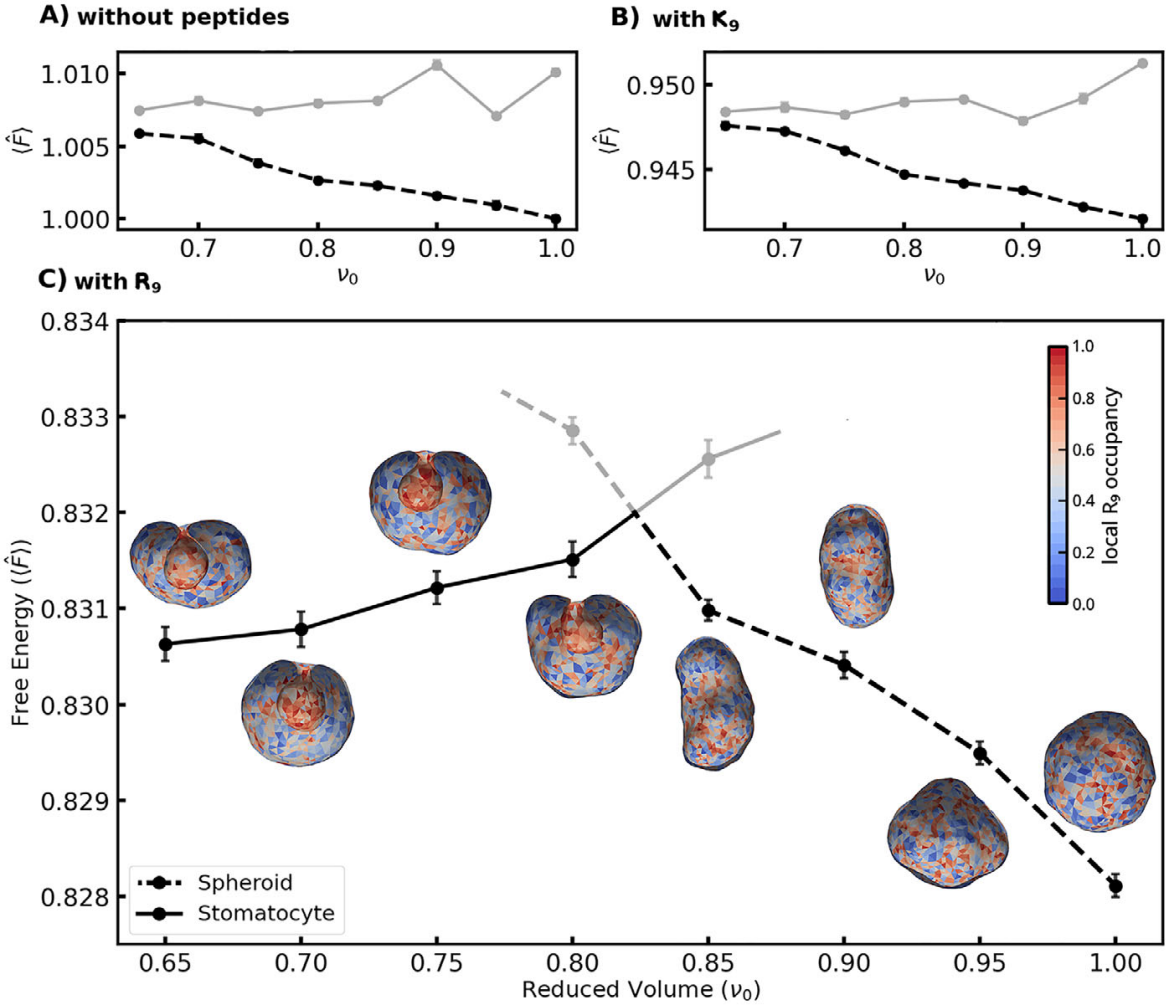
Model free energies. All energies are given relative to the spherical DOPE:DOPC:DOPS system at $\nu_{0}=1.0$ without peptides. Relative stability of spheroids vs stomatocytes is given: A: without peptides, B: with $K_{9}$, and C: with $R_{9}$. Simulation snapshots depict the corresponding reduced volume, with color maps indicating average sorting occupancy. Grey plots denote thermodynamically unstable states. Error bars represent the 95% condfidence interval.

We compare its relative energy to that of prolate/oblate spheroid‐type structures in Figure 6. Since our Monte Carlo simulations sample the Boltzmann distribution, high‐energy states have negligible equilibrium population (i.e. curves marked in gray are thermodynamically unstable shapes that tend to transition toward the lower stable states in $∼8\times 10^{6}$ MC steps). Due to the high number of MC steps required for a transition, these unstable states can be sampled in the same way as the stable geometries to estimate relative energies (see Methods). Stomatocyte shapes are unstable with respect to prolate geometries in the simulations without peptides (see Figure 6A). This behavior mirrors the predicted global minimum resulting from Helfrich energy minimization without $J_{s}$. Hence, our result aligns with the community's established understanding that ideal mixing entropy prevents lipid demixing to a large extent [48, 73] as the spontaneous curvatures of monolayers cancel out for opposite membrane leaflets of identical composition.

The energy profiles of membranes containing $K_{9}$ peptides (shown in Figure 6B) are similar to the control run in that *no stable* range for the stomatocyte structure is observed for $\nu_{0}>0.65$.

**FIGURE 7:**
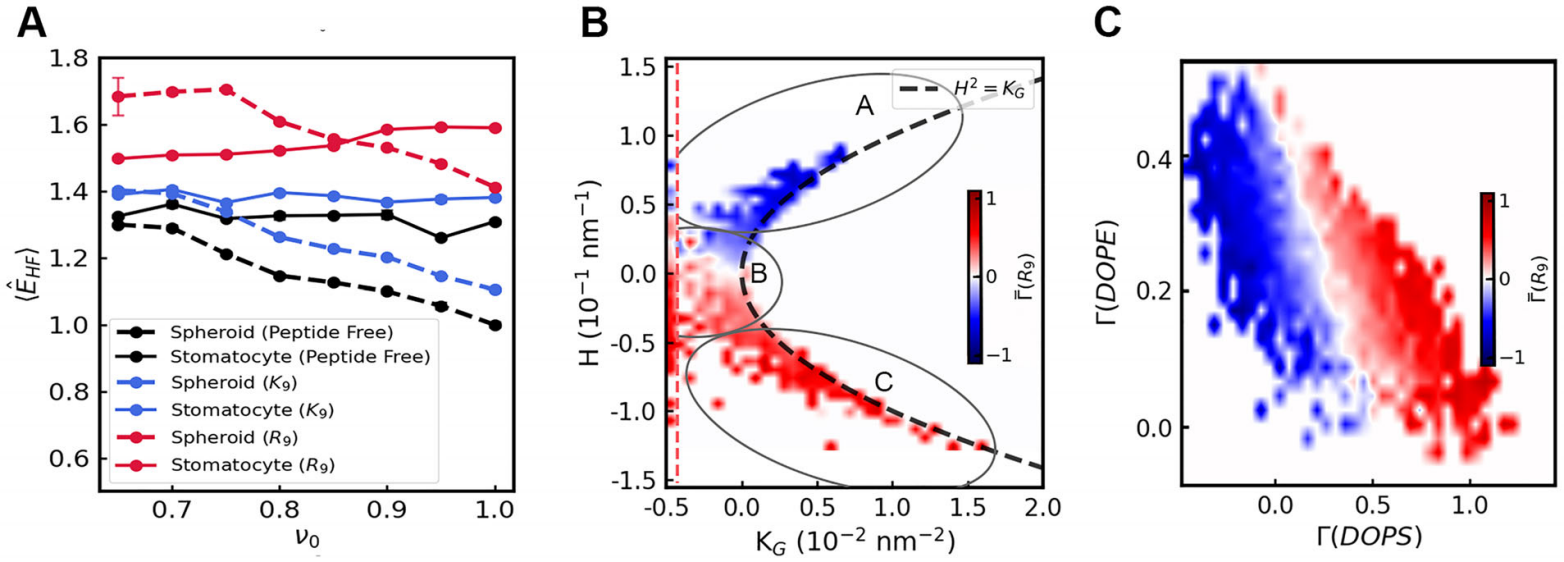
(A) Contributions of curvature elastic term (Helfrich energies). Profiles are shown under three conditions: peptide‐free system (black), with $K_{9}$ (blue), and with $R_{9}$ (red). Dashed lines represent spheroid morphologies, while solid lines indicate stomatocyte morphologies. Error bars denote the 95% confidence interval. All energies are given relative to that of the spherical DOPE:DOPC:DOPS system at $\nu_{0}=1.0$ without peptides. (B) Bin‐averaged heatmap of excess $R_{9}$ coverage on stomatocyte at reduced volume ($\nu_{0}$) of 0.80. Regions A, B, and C correspond to the exterior, neck, and invagination, respectively. The dashed line delineates the excluded region by binning out all outliers to improve data clarity. The dashed line $H^{2}=K_{G}$ marks the geometric boundary of real surfaces, and corresponds to a perfectly spherical (or planar) shape where curvature is isotropic. (C) Heatmap showing the excess coverage of $R_{9}$ as a function of DOPE and DOPS composition.

The presence of $R_{9}$ peptides significantly enhances the stability of invaginated structures compared to (prolate) spheroids. Energetically, invaginated and prolate shapes were comparable within a reduced volume ($\nu_{0}$) range of 0.80−0.85. Below $\nu_{0}\approx 0.80$ stomatocyte geometries have considerably lower energy than spheroids, in contrast to what is observed in the peptide‐free simulation or the simulation with $K_{9}$. This stabilization is driven by the spontaneous curvature generation of $R_{9}$, as evidenced by the curvature energy contribution (see Figure 7A). This plot also reveals that the thermodynamic stability of the invagination decreases with a reduction in available membrane area, the osmotic pressure progressively destabilizes this geometry as the vesicle is “filled‐up”. A detailed energy decomposition plot is provided in Figure S2.

Collectively, these findings indicate that $R_{9}$ peptides stabilize inward budding in DOPE:DOPC:DOPS vesicles, but require a minimum excess area or hyperosmotic stress. The stabilization of these shapes requires spontaneous curvature generation by $R_{9}$, concomitant with significant lipid demixing, which is driven by curvature sorting, but particularly the differences in peptide binding free energies between lipids.

#### 3.4.2 Curvature Sorting

In order to quantify curvature sorting, we define the *fractional excess coverage* as:

(20) $$\bar{\Gamma }\left(i\right)=\frac{n^{\left(i\right)}}{n_{0}^{\left(i\right)}}-1$$

Here, $n^{\left(i\right)}$ is the occupancy (number of lipids or peptides) of type i on a face, and $n_{0}^{\left(i\right)}$ is the amount of the $i^{\text{th}}$ compound according to the global composition. Figure 7B,C displays heatmaps of the fractional excess $R_{9}$ coverage on a fixed mesh representing a typical “stomatocyte” or inward‐budded vesicle with $\nu_{0}=0.80$, calculated by first binning the instantaneous mesh data to preserve spatial correlations and averaging over the last 200 such snapshots.

The excess coverage $\Gamma (R_{9})$ is analyzed as a function of mean curvature (H) and the Gaussian curvature ($K_{G}$) in Figure 7B. Region A, corresponding to the vesicle exterior (H>0, $K_{G}>0$), shows a depletion of $R_{9}$. In contrast, Region B, representing the invagination neck (H∼0, $K_{G}<0$), and Region C, representing the interior of the bud (H<0, $K_{G}>0$), exhibit a significant enrichment of $R_{9}$.

This noticeable $R_{9}$ localization to the invagination and neck regions is accompanied by a significant redistributions of DOPE and DOPS lipids, as illustrated in Figure 7C. These lipids exhibit pronounced negative curvatures upon interaction with $R_{9}$. This sorting illustrates the role of lipid‐peptide interactions in driving curvature generation. The Helfrich energy decomposition (see Figure S2) reveals distinct driving forces for this sorting: while the accumulation in the invagination is driven by the relaxation of the dominant mean curvature energy, the accumulation in the neck is locally driven by small mean curvature. This effect might even be underestimated, as direct calculations of ${\bar{\kappa }}_{b}$ reveal potentially strong negative Gaussian curvature generated by $R_{9}$ (see Table S1). We verified the robustness of our results against various values of $\bar{\kappa }$ including zero, hence an important role of $\bar{\kappa }$ is unlikely. In our simulations, the binding energy of peptides to lipids also mediates the induced lipid sorting. $R_{9}$ molecules have a lower mixing entropy than the lipids and cover multiple lipids by one peptide. In our model, the presence of $R_{9}$ comes without a *per face* internal mixing entropy. Nevertheless, the expansion of $R_{9}$ on its mesh lattice sites is associated with its own entropy, reflected by its wide distribution. This entropy emerges from generating a Monte Carlo scheme, which distributes the peptides on the mesh. Yet, this “moving lipid bracket” formed by the peptide facilitates the symmetry breaking necessary for bud stabilization.

## 4 Conclusion

The chemical specificity of lipid bilayer interaction with the efficient cell‐penetrating peptide $R_{9}$ over $K_{9}$ is visible both in the binding energies and in the curvature generation. $R_{9}$ binds more strongly and generates more *negative* curvature than $K_{9}$ for all examined lipids. In contrast to other studies, we find this effect to be unrelated to guanidinium pairing. Instead, the deeper penetration of the guanidinium sidechains into the headgroup region of the membrane is responsible for the observed differences.

We systematically transferred these specific interactions into effective parameters of a continuum model. After validating this model, we used it to elucidate the mesoscopic *consequences* of chemical specificity.

Inward budding is believed to be the initial step of cell penetration [7]. Our mesoscopic, curved‐element DTS simulations show how the $R_{9}$ peptide stabilizes inward buds. These invaginations exhibit a strong tendency for $R_{9}$ localization in regions of negative mean and negative Gaussian curvature. The formation of such structures requires sufficient available membrane, corresponding to a reduced volume of approximately 0.8−0.85, thereby requiring either an excess membrane area or the application of hyperosmotic stress to induce the transition. This poses a challenge for lipid vesicle experiments, but not for cells with rough membrane surfaces. $K_{9}$ was found to be unable to generate this type of structure.

Our findings indicate that strong binding and curvature generation are key to $R_{9}$ cell penetration. The crosslinking and membrane‐aggregating effect of $R_{9}$ on membranes is the known unknown in the mechanism of CPP entry. Finally, these results do not contradict our previous investigations [3] in any way, as multilamellarity and fusion are expected to arise in subsequent steps.

## 5 Methods

### 5.1 Molecular Dynamics Simulations

A bilayer containing 1024 lipids per leaflet was built by CHARMM‐GUI [74] containing DOPE:DOPS:DOPC lipids (60:20:20). 64 peptides, 50 TIP3P water molecules per lipid, and 150mM KCl plus additional ions to counteract peptide charges were added. We performed simulations in Gromacs [75] for 1μs while the last 200ns were used for analysis. Simulations were performed in an NpT ensemble, using Nosé–Hoover [76, 77] temperature coupling with a 1ps time constant and semi‐isotropic Parrinello–Rahman [78] pressure coupling (1bar). We used Particle‐Mesh Ewald electrostatics [79] with a cutoff of 1.2nm. Hydrogen atom bonds were constrained by LINCS [80]. The simulation timestep was set to 2fs.

#### 5.1.1 Material Properties

The simulated systems were single lipid membranes, containing 14000 TIP3P water molecules and 128 lipids, in addition to 150mM ions (and neutralizing counterions in the case of DOPS). When adding peptides, we added 14 $R_{9}$ or $K_{9}$ to DOPS and 6 $R_{9}$ or $K_{9}$ to DOPC and DOPE, respectively, for the simulation of a fully covered membrane. We also ran a 6 $R_{9}$ partially covered membrane. The simulations were run in the presence of 150mM ions and counterions. For the stress tensor [27, 45] and ReSiS [36] extraction simulations, we used the unscaled CHARMM36m. For the computation of elastic properties, simulations of pre‐equilibrated bilayers were continued for 200ns using Gromacs 2020.3 [75]. We simulated at a temperature of 300K, using the same ensemble, algorithms, and cutoffs as in the previous section. Long‐range electrostatics were treated using the particle‐mesh Ewald method [79]. Since the pressure decomposition does not support the SETTLE algorithm [81], the triangular geometry of water was constrained using LINCS with order five [80]. No dispersion corrections or potential switching were applied. Further simulation details for stress‐tensor computations and details for the free energy profiles are provided in the Supporting Information.

### 5.2 Mesoscopic Monte Carlo Simulations

Metropolis MC simulations were performed using OrganL [29], our DTS code. The model implementation for this paper will be uploaded here.

#### 5.2.1 System Initialization and Shape Generation

Osmolyte concentration $c_{0}$ was set to 300mM (300mOsm) in agreement with standard PBS buffers, and the area compressibility modulus $K_{A}$ was set to 200pN/nm [82, 83]. We initialized the system with a uniform lipid composition of DOPE:DOPC:DOPS 60:20:20 and a protein coverage of $\phi_{p}=0.5$, with parameters detailed in Table S3. At this coverage (50%), all PS lipids are neutralized, along with a fraction of PE lipids. The bound peptide‐to‐lipid ratio is 1:46. We estimated it using our computed adsorption energies and the empirical data [11].

The simulation was initialized with an equilateral spherical mesh of radius R=50nm, discretized into 2152 faces. This discretization was chosen such that the average area per face matches the projected area of an $R_{9}$ peptide. To generate the initial stomatocyte geometries used for sampling, we employed a specific shape‐annealing protocol starting from the spherical mesh ($\nu_{0}=1$). A stable stomatocyte geometry was achieved over approximately $3\times 10^{5}$ MC steps by setting the target reduced volume to

$$\nu_{0}=1/\sqrt{2}\approx 0.707$$

effectively modeling the geometry of two fused equal‐sized vesicles. The Monte Carlo step sizes were set to 1.0 for both vertex displacement and vertex normal displacement, with autotuning over the first 10 steps to attain good acceptance ratios (∼0.3 for Vertex moves, ∼0.7 for Normal moves and ∼0.8 for Lipid moves) for the MC steps [29].

Achieving a realistic narrow‐neck stomatocyte required manipulating the volume constraint. The stomatocyte was simulated with the target volume $V_{0}$ iterating back and forth near $\nu_{0}\approx 0.85$, coupled with a high frequency of bond‐flipping moves (remeshing every 500 MC steps with 10 iterations), as necessary for constriction. This geometry served as the starting point for all subsequent stomatocyte simulations (control, $K_{9}$, and $R_{9}$), while the initial equilateral spherical mesh is used as the starting geometry for the spheroids.

#### 5.2.2 Volume Scanning and Convergence

To compute the energy profile as a function of reduced volume (Figure 6), we performed a hysteresis scan. Starting from the stabilized stomatocyte, the target volume V was gradually increased back toward the initial spherical value ($\nu_{0}\to 1$). At each step, the system was sampled until the standard deviation of the instantaneous reduced volume ν was below <0.01. This typically required an equilibration phase of $∼2\times 10^{6}$ MC steps per geometry.

#### 5.2.3 Data Analysis and Averaging

Following the initial equilibration phase, another $∼1\times 10^{6}$ run is performed and snapshots sampled every 1000 MC steps are selected for analysis. This sampling window is chosen because transitions to more stable branches are typically observed in $∼8\times 10^{6}$ steps. By sampling at this interval, we capture the most representative state of the current geometry before long‐term fluctuations occur. To improve computational efficiency, we employed a relatively lower frequency of bond—flipping moves at every 1200 integration steps, using 10 iterations per remeshing cycle. Mesh configurations were recorded every 1000 steps to track the evolution of the system.

- **Energy Calculation**: System energies are computed by averaging over 1000 outputs, corresponding to the last $10^{6}$ Monte Carlo steps.
- **Mesh Averaging (Visualization)**: To generate the representative structures shown in Figure 5, we applied a *mesh averaging* procedure using the Iterative Closest Point (ICP) algorithm [84] over the last 200 output files. The face properties are averaged and mapped onto this ICP‐generated mesh to produce the representative images included in the manuscript.
- **Bin Averaging (Visualization)**: To preserve spatial correlations for curvature analysis (e.g., distinguishing high‐curvature neck regions from the bulk), we employed a *bin‐averaging* approach on the last 200 snapshots (200k MC steps). In this method, a local property (e.g., lipid density) is binned according to a second variable (e.g., mean curvature), and values are averaged within these bins across all snapshots. This ensures that correlations between local geometric features and chemical composition are not lost due to the spatial averaging process.

The error bars reported in the continuum modeling study represent the standard error on the mean corrected for temporal correlations inherent in Monte Carlo sampling [85]. To account for these correlations, we calculated the statistical inefficiency, s of total energy across ∼50k points spanning $∼1\times 10^{6}$ MC steps, by integrating the normalized autocorrelation function, C(k), of the total energy up to its first zero‐crossing (automatic windowing). This factor s quantifies the number of steps required to generate one effectively independent sample. The true standard error was subsequently derived by rescaling the naive error estimate by $\sqrt{s}$, according to $\sigma_{\text{true}}=\sigma \sqrt{s/N}$, where σ is the population standard deviation and N is the total number of samples. Further, a 95% Confidence Interval (CI) on the mean is attained by multiplying this value by a z‐score of 1.96.

## Author Contributions

CA designed and supervised the research. KB and CA performed MD simulations and their analysis. JK performed DTS simulations and analysis. CA and JK implemented the model. CA, JK, and KB wrote the paper.

## Conflicts of Interest

The authors declare no conflicts of interest.

## Supporting information


**Supporting File**: smtd70603‐sup‐0001‐SuppMat.pdf.

## Data Availability

The source code of the model will be made available on GitHub after publication. The underlying data for this study are available from the author upon reasonable request.
