# Supplementary material for: From Molecular Insight to Mesoscale Membrane Remodeling: Curvature Generation by Arginine‐Rich Cell‐Penetrating Peptides
Source: Small Methods. 2026 Mar 24;10(8):e01539. doi: 10.1002/smtd.202501539 (PMC13103641; doi:10.1002/smtd.202501539)
Supplement: Supplementary file 1 — Supporting File: smtd70603‐sup‐0001‐SuppMat.pdf. [file SMTD-10-e01539-s001.pdf]

Supporting Information - Multiscale Modelling and Chemical Specificity of Lipid Membranes; Case of Arginine Magic

Katarína L. Baxová, Jovi Koikkara, Christoph Allolio

1 Arginine-Arginine Interaction in Molecular Dynamics

Our molecular dynamics (MD) simulations did not show R_9 peptides to self-aggregate while interacting with lipid bilayers. We calculated two-dimensional R_9-R_9 radial distribution functions (RDFs) in a plane parallel to the membrane (the x-y plane of the box) for arginines interacting with the upper and lower membrane leaflets, separately, using the last 200 ns of simulations. Figure S1 shows the average of these two RDFs, with the calculated error both for CHARMM and (scaled) ProsECCo.

We found no significant peptide aggregation throughout the simulation, both when using the CHARMM force field and the charge-scaled ProsECCo parameters. [1]

2 Parameter Extraction from Molecular Dynamics

2.1 Stress Tensor

The local stress tensor was calculated using the rerun functionality of the gmx mdrun module of our implementation [2] of a Goetz-Lipowsky-Decomposition [3] into the code by Sega *et al.* [4] The modified version of GROMACS is available at `https://github.com/allolio`, delivered to be compatible with M. Sega's code. Our approach is similar to the method in Ref. [5]. Specifically, we set the normal pressure p_N to impose zero surface tension:

$$\sigma = \int_{-l}^{l} \pi(z)\,\mathrm{d}z = \int_{-l}^{l} \Big[-\frac{1}{2}\{p_{xx}(z) + p_{yy}(z)\} + p_\mathrm{N} \Big]\,\mathrm{d}z = 0. \tag{1}$$

Here, σ is obtained from the diagonal components of the stress tensor [6], and the integral is carried out over the entire simulation box $[-l, l]$ along the membrane normal z, where $z = 0$ corresponds to the bilayer center. Since this approach requires p_N being close to 1 bar, we validated that the deviations from this target were ≤ 4.1 bar for all simulations. We used at least 20000 snapshots which included velocities for the rerun. The rerun was done using rebuilt neighbor lists for each frame.

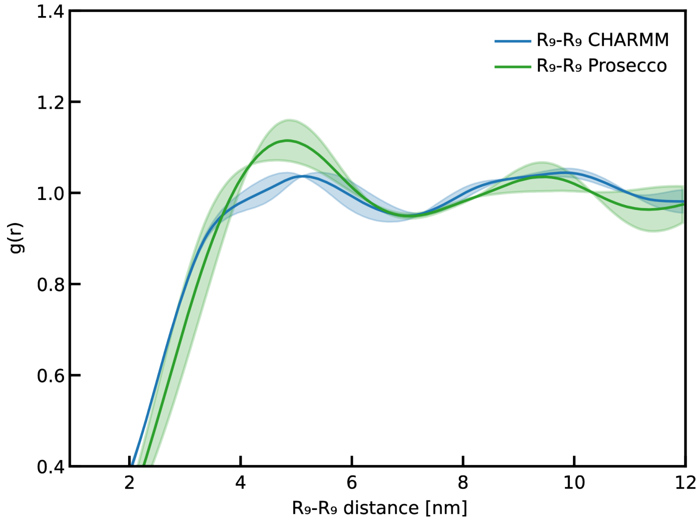

Figure S1: Arginine self-aggregation: two-dimensional RDF of R_9-R_9. It was computed as the average of a 2D R_9-R_9 RDFs obtained from nonaarginines on the upper and lower membrane leaflet, with calculated error. Both CHARMM and ProECCo force fields were used.

2.2 Extraction of Model Parameters

The bending modulus κ and the tilt modulus κ_θ were calculated using the ReSIS method. [7] We used lipid director definitions and an implementation of the LIPIDATOR-TOOLKIT available at `https://github.com/allolio/lipidator-toolkit`. This allowed the use of the well-known relation [8, 9] of the first bending moment to the product of spontaneous curvature, J_s and κ:

$$\kappa J_\mathrm{s} = \int_0^l \pi(z) z \mathrm{d}z. \tag{2}$$

Here, the integration is carried out along the membrane normal $\hat{\mathbf{e}}_z$ and only over one monolayer, and, therefore, monolayer elastic properties are used. The reported values are averages over both monolayers. We used Gaussian error propagation obtained from subsets of the data (sequential 3 samples for the stress profile, 10 samples from the ReSIS calculation).

2.2.1 Definition of Lipid Excess

The number of lipids bound to the peptide was extracted from the scaled RDF data from MD using the 2D Kirkwood Buff Integral:

$$\Gamma = \sigma_\beta \int 2\pi r (g_{\alpha\beta}(r) - 1) \mathrm{d}r, \tag{3}$$

where σ_β is the surface density of β-particles. The RDF values used are the averages of "Charmm" and "ProsECCo" results.

Lipid	Direct	Synthetic
DOPS	-18.15	-11.128
DOPS + 14 R_9	9.79	-9.28
DOPS + 6 R_9	-14.19	-10.872
DOPS + K9	4.1	-12.192

Table S1: Bilayer Gaussian Bending Moduli (all units in kT).

2.3 Binding Energies

The binding energies were computed via Umbrella sampling at 300 K, the setup used 128 lipids (either DOPE, DOPC, or DOPS), 14K H_2O molecules, and 150 mM KCl ions, in addition to neutralizing counterions. This corresponds to 34 K^+ and 43 Cl^- for neutral membranes and 153 K^+ with 34 Cl^- for DOPS. After pre-equilibration, each umbrella window was run for 250 ns. The pull coordinate was the center of mass distance along the membrane normal, between lipids and peptides. The force constant was typically 1000 kJ/mol/nm^2, and 21 windows were used for each simulation. The binding energies were computed using a charge scaling derivative of the CHARMM36m/CHARMM36 force field - ProsECCo75 [1] with the TIP3P water model.

2.4 Notes on the Gaussian Bending Modulus

From the very beginning the Gaussian Bending Modulus was regarded as problematic. [10] Here we compare computed Gaussian Bending moduli between the extrapolation scheme and direct stress tensor computations at the case of R_9 and K_9 on DOPS. Results are given in Tab. S1. Note that we do not give any error estimates, as we have low confidence in providing errors for the direct calculation.

3 The Mesoscopic Model

The simulations use the OrganL package. [11] We describe how peptides bind to bilayers composed of mixed lipids and influence the membrane properties like intrinsic curvature J_S or bending rigidity κ, as defined in the Canham-Helfrich [12, 13] free energy

$$F_{\mathrm{HF}} = \int_{\mathrm{Surf}} \mathrm{d}A \left\{ \frac{\kappa}{2} (2H - J_S)^2 \right\}. \quad (4)$$

For this purpose, we construct a model describing the curvature influence for arbitrary mixtures (as we assume that lipid demixing occurs). In other words, κ and J_S can vary locally. We also want to simulate the vesicle geometries under realistic osmotic pressure and surface tension conditions. The protein binding model closely resembles that of a **regular solution**, i.e. we will assume the ideal mixing of lipids (a standard assumption) together with a fixed, incremental binding energy of the peptide up to the maximum concentration. To support the latter assumption, we note that according to Cremer et al. [14], the binding of R_9 in particular is noncooperative, i.e., modelled well by a Langmuir isotherm, which implies noninteracting binding sites.

3.1 Effect of Lipids

3.1.1 Monolayer

The mixing rules are commonly found in the literature and summarized here from [11], where this implementation was originally published. We calculate the **bending modulus** as a harmonic average of the single lipid moduli κ_i:

$$\frac{1}{\kappa} = \sum_i \phi_i \frac{1}{\kappa_i}. \tag{5}$$

Here, ϕ_i refers to the volume fraction, which is calculated as

$$\phi_i = \frac{n_i A_i}{\sum_j n_j A_j}, \tag{6}$$

with A_i denoting the area per lipid (APL) of the lipid species i. We assume that mixing does not change the area per lipid. The APL A_i is, hence, a reference area contributing to A_0. By n_i, we refer to the number of lipids of species i present in a triangle.

The spontaneous curvature is computed as

$$J_s = \sum_i \phi_i c^0_{s,i}, \tag{7}$$

where c^0_i is the **spontaneous curvature** of pure i. [15, 16]We have recently tested the validity of these equations against atomistic molecular dynamics results. [5] Furthermore, the **mixing entropy** of the lipid composition with a total occupancy of lipids M on a side/leaflet of a triangle can be estimated as ideal in the surface fractions [15]:

$$F_{\text{mix}} = kTM \sum_i \phi_i \ln \phi_i. \tag{8}$$

3.1.2 Bilayer

For the averaging of the two sides, we use the indices u, d for the upper and lower monolayers, respectively, and b for the bilayer:

$$\kappa_b = \kappa_u + \kappa_d;$$
$$F_{\text{mix,bilayer}} = F_{\text{mix,u}} + F_{\text{mix,d}} \tag{9}$$

The spontaneous curvature is computed as:

$$J_{\text{s,bilayer}} = \frac{1}{2}\big(J_{s,u} - J_{s,d}\big). \tag{10}$$

The sign change is due to a normal orientation and curvature direction change when flipping between two faces. The resulting equations are equivalent to averaging over two monolayers, up to a constant offset, under the condition that $\kappa_u \approx \kappa_d$ and the thickness $d \to 0$. The error bars for κ and κ^b_θ were obtained by Gaussian fitting to the tilt and the divergence of the tilt distribution, respectively. Ten separate distributions were computed by sampling independent parts of the simulation trajectory; the standard deviations from values thus

obtained were used to calculate the standard error over the mean of the entire trajectory. In the case of J_s, the monolayers were averaged, and three windows were used to compute the standard error for $J_S\kappa$. We computed Gaussian error propagation to obtain the error bars.

3.2 Effect of Peptides

The peptides are assumed to occupy an area A_p, *on* a lipid monolayer. Then, the coverage of the peptide is computed by

$$\phi_p = n_p \frac{A_p}{A_l}, \tag{11}$$

where n_p is the number of peptides. ϕ_p is controlled not to exceed 1. A_l is the total area of the lipid patch, i.e.

$$A_l = \sum m_i A_i, \tag{12}$$

which is calculated on a leaflet. For each pure lipid i, there is a binding free energy μ_i with the peptide. Accordingly, the total binding energy of the peptide is estimated as

$$F_b = n_p \sum_i \phi_i \mu_i. \tag{13}$$

The influence of the peptide on the lipid membrane properties is further evaluated by analyzing the lipid surface fractions within the membrane patch. Each pure lipid species interacting with the peptide contributes its respective bending rigidity, κ_i, which differs from the value observed in the absence of the peptide. The net value is computed as:

$$\frac{1}{\kappa_p} = \sum_i \phi_i \frac{1}{\kappa_i} \tag{14}$$

and finally weighted as

$$\frac{1}{\kappa} = \phi_p \frac{1}{\kappa_p} + (1 - \phi_p)\frac{1}{\kappa_n}, \tag{15}$$

where κ_n is the bending rigidity computed without peptide influence as in the previous section.

Similarly,

$$J_{s,p} = \sum_i \phi_i c0_i \tag{16}$$

and

$$J_s = \phi_p J_{\mathrm{s,p}} + (1 - \phi_p) J_{\mathrm{s,n}}. \tag{17}$$

Note that the peptide does not contribute to the mixing entropy, as it is considered large with respect to the mixture. The number of peptides is always an integer, but the number of lipids can be a rational number on each face.

3.3 Gaussian Curvature

In the absence of a good estimator for the Gaussian curvature, the bilayer Gaussian curvature is computed as [2, 17]

$$\bar{\kappa}_b = -0.8\kappa_b - 2J_{s,b'}\kappa_b d, \tag{18}$$

which is a result of an expansion to linear order in the membrane thickness from bilayer midplane d, taken to be 1.2 nm. The prefactor of 0.8 was taken by comparing monolayer Gaussian curvature moduli measured from the literature [2]. To calculate $J_{s,b'}$, monolayer J_s were added instead of subtracted (as monolayer curvatures do not cancel in this calculation).

3.4 Relating Bilayer Properties to Monolayer Properties

We derive the effective elastic moduli of the bilayer by treating it as two coupled monolayers separated by a fixed distance 2δ. We assume the thin-shell limit ($|\delta J| \ll 1$), approximating area dilation factors as unity ($A_{\text{out}} \approx A_{\text{in}} \approx A$), as in the derivation by Siegel and Kozlov[18].

3.4.1 Geometric Relations

Let J and K denote the total and Gaussian curvatures of the bilayer mid-plane. The curvatures of the outer and inner monolayers, expanded to linear order in δ, are given by:

$$J_{\text{out}} \approx J + 2K\delta, \qquad K_{\text{out}} \approx K, \tag{19}$$

$$J_{\text{in}} \approx -J + 2K\delta, \qquad K_{\text{in}} \approx K, \tag{20}$$

where the sign inversion in J_{in} reflects the opposing normal of the inner monolayer relative to the mid-plane.

3.4.2 Energy Expansion

The total bilayer energy density f_b is the sum of the Helfrich energies of the monolayers:

$$f_b = \sum_{i\in\{\text{out, in}\}} \left[\frac{1}{2}\kappa_i(2H_i - J_{S,i})^2 + \bar{\kappa}_i K_i\right]. \tag{21}$$

Substituting Eq. (1) and (2) into Eq. (3) and retaining terms up to $\mathcal{O}(\delta)$, we match the resulting coefficients to a generalized bilayer Hamiltonian of the form:

$$f_b = \frac{1}{2}\kappa_b(2H - J_{S,b})^2 + \bar{\kappa}_b K_G + C_{\text{mix}} H K_G + \text{const.} \tag{22}$$

The resulting relationships between bilayer and monolayer parameters are summarized in Table S2. In the limit of a symmetric bilayer, these expressions recover the standard relations derived by Siegel and Kozlov[18]. The constant term constitutes a surface tension and is considered to be implicitly included in the area compressibility term.

We observe from Table 3 that the weighted bending modulus (κ) for the 60 : 20 : 20 DOPE:DOPS:DOPC system remains nearly constant upon the addition of R_9 (14.66 vs. 14.77 k_BT). Given this negligible difference, we simplify

Property	Expression	Small $\Delta\kappa$
Bending Modulus (κ_b)	$\kappa_{\text{out}} + \kappa_{\text{in}}$	$\kappa_{\text{out}} + \kappa_{\text{in}}$
Spont. Curvature ($J_{S,b}$)	$(\kappa_{\text{out}} J_{\text{S,out}} - \kappa_{\text{in}} J_{\text{S,in}})/\kappa_b$	$(J_{\text{S,out}} - J_{\text{S,in}})/2$
Gaussian Modulus ($\bar{\kappa}^b$)	$\bar{\kappa}_{\text{sum}} - 2\delta(\kappa_{\text{out}} J_{\text{S,out}} + \kappa_{\text{in}} J_{\text{S,in}})$	$\bar{\kappa}_{\text{sum}} - \delta\kappa_b(J_{\text{S,out}} + J_{\text{S,in}})$
Coupling Modulus (C_{mix})	$2\delta(\kappa_{\text{out}} - \kappa_{\text{in}})$	0

Table S2: Effective bilayer moduli derived from monolayer properties. In the small $\Delta\kappa$ approximation, $\kappa_{\text{out}} \approx \kappa_{\text{in}}$.

the calculation of the bilayer spontaneous curvature (J_S^b) and Gaussian curvature ($\bar{\kappa}_b$) by assuming a small difference in bending modulus across the leaflets ($\kappa_{\text{out}} \approx \kappa_{\text{in}}$). If additionally $J_{\text{S,out}} = J_{\text{S, in}} = J_{\text{S}}$, the relations by Siegel and Kozlov are recovered.

For completeness, the full formulation—allowing distinct inner and outer leaflet moduli—is also implemented in our code. This extended model can be activated by setting `EffectiveLipidModel` to 1 in control.txt, as described in the organL manual. This implementation neglects C_{mix} as it is of third order in principal curvature and was checked to be small. Note that this model allows for large variations κ, but not in δ.

3.5 Total Energy

The total energy of the system is then given by

$$F = \int_{\text{Surf.}} \mathrm{d}A \left\{ \frac{\kappa_b}{2}(2\tilde{H} - J_{S,b})^2 + \bar{\kappa}_b K_G \right\} + E_{\text{pV}} + E_{\text{stretch}} + \sum_{\text{face}} \left[F_b + F_{\text{mix,b}} \right] \tag{23}$$

In this equation, the parameters $\kappa, \bar{\kappa}$ and J_S are constant on each face as far as integration is concerned. The integration is performed over the faces. F_b and $F_{\text{mix,b}}$ are computed per face and summed up. E_{pV} and E_{stretch} are computed using global values for (A, A_0) and (V, V_0), which are the (current, initial) values, respectively.

3.5.1 Area and Volume Control

The film area is controlled via the area compressibility K_A, and the following stretching energy is added to the total system:

$$E_{\text{stretch}} = \frac{K_A}{2} \frac{(A - A_0)^2}{A_0} \tag{24}$$

We use the isothermal ideal gas equation of state to relate osmotic pressure to the vesicle volume:

$$\Delta p = \Delta c R T. \tag{25}$$

Assuming that the vesicle is in equilibrium with the external pressure, the isothermal volume work, relative to the equilibrium volume V_0, which defines the reference state where $E_{\mathrm{pV}} = 0$, can be expressed as

$$E_{\mathrm{pV}} = c_0 k_B T \big[V - V_0 - V_0 \log(|V/V_0|) \big] \tag{26}$$

where c_0 is concentration, V_0 is the initial volume, k_B is the Boltzmann constant, and T is the absolute temperature.

3.6 Model Validation: Optimization

For the optimization, we considered a flat membrane divided into two virtual membrane systems: one functionalized with a peptide and the other unmodified. The free energy concerning lipid and protein is

$$\begin{aligned} F = kTM_f \sum_i \phi_{i,f} \ln \phi_{i,f} \\ + M_p \sum_i \big[kT\phi_{i,p} \ln \phi_{i,p} + \phi_{i,p}\mu_{i,p} \big]. \end{aligned} \tag{27}$$

Since the membrane is flat, the bending terms will not affect the mixing. We also approximate $\forall i, j : A_i = A_j \Rightarrow \phi_i = x_i$, so we can express the energy in mole fractions x_i. We divide a lipid population into a free fraction M_f and a lipid population M_p bound to the protein with a binding energy $\mu_{p,i}$. The model Hamiltonian can, therefore, be expressed as:

$$\begin{aligned} F \approx kTM_f \sum_i x_{i,f} \ln x_{i,f} \\ + M_p \sum_i \big[kT x_{i,p} \ln x_{i,p} + x_{i,p}\mu_{i,p} \big] \end{aligned} \tag{28}$$

We also apply the following constraints:

$$M_f x_{i,f} + M_p x_{i,p} = M x_i, \quad \forall\ x_i \in (0,1) \tag{29}$$

$$\sum_i x_{i,f} = 1 \quad \text{or} \quad \sum_i x_{i,p} = 1 \tag{30}$$

$$x_{\mathrm{DOPE}} = 0.6,\ x_{\mathrm{DOPC}} = 0.2,\ x_{\mathrm{DOPS}} = 0.2 \tag{31}$$

where $M := M_f + M_p$ is the total number of lipids and x_i is the mole fraction of ith lipid, both of which are fixed. Applying either condition in the Eq. (30), in conjunction with Eq. (29), inherently satisfies the complementary constraint. The resulting energy is minimized against Eq. (28) for a given M_p. Since the M_p value depends on the unknown peptide area, M_p is an adjustable parameter. We optimize it by minimizing a $\mathcal{L}^2$ norm between the MD data (Table 1 of manuscript) and the estimated $\{x_{i,p}\}$ after converting the latter to excess quantities. The technical details are as follows:

3.6.1 Other Details

The optimization procedure consists of two sequential sub-problems:

(a) No peptide

no peptide	κ $[kT]$	c^0 $[nm^{-1}]$	A_0 [nm]	E_{bind} [kJ/mol]
DOPE	15.83	-0.24	0.625	×
DOPS	14.27	-0.09	0.643	×
DOPC	11.56	0.0	0.679	×

(b) With R_9

R_9	κ $[kT]$	c^0 $[nm^{-1}]$	A_0 [nm]	E_{bind} [kJ/mol]
DOPE	16.25	-0.30	0.625	-23.2 ± 2.6
DOPS	13.60	-0.25	0.643	-86.0 ± 4.3
DOPC	10.88	-0.04	0.679	-19.6 ± 3.0

(c) With K_9

K_9	κ $[kT]$	c^0 $[nm^{-1}]$	A_0 [nm]	E_{bind} [kJ/mol]
DOPE	15.83	-0.24	0.625	-0.7 ± 1.3
DOPS	16.26	-0.17	0.643	-51.4 ± 2.3
DOPC	11.56	0.0	0.679	×

Table S3: Summary of model parameters for lipid bilayers with/without peptides[2].

1. **Free Energy Minimization:** The free energy functional, defined in Eq. (28), is minimized subject to the constraints specified in Eqs. (29–31). This constrained optimization is carried out using the `trust-constr` algorithm (default `SLSQP` doesn't converge for K_9) from the `SciPy` optimization library, which supports the inclusion of `LinearConstraint` objects.

2. **Fitting to Simulation Data:** The above minimization is wrapped using the $\mathcal{L}^2$ norm of the discrepancy between the predicted excess quantities and those obtained from molecular dynamics simulations (as described in the main text) and is minimized. This step is implemented using the `Powell` method available in `SciPy`.

3.7 Mesoscopic Monte Carlo Simulations

The model parameters used for the simulations are summarized in Table S3.

The starting point for the simulation was an equilateral spherical mesh of radius 50 nm with 2152 faces (so that the area per face matches the (R9) area).

A stable stomatocyte geometry was generated from the spherical mesh in $\approx 3 \times 10^5$ monte carlo (MC) steps (with step size of 1.0 for both `VertexMove` and `NormalMove`; and `autoTune` of 10 steps) [11] by setting the reduced volume

$$\nu_0 := \frac{6V}{(A)^{3/2}}\sqrt{\pi} = \sqrt{2}^{-1}. \tag{32}$$

This corresponds to the situation of two vesicles of equal size having fused, i.e. doubling A and V from an initial spherical geometry ($\nu_0 = 1$). This

was achieved by reducing V while keeping A constant. To get a stomatocyte structure with a good constriction at the neck, it is necessary to simulate back and forth by changing the values of V_0 at ~ 0.85, with high `Remesh` values [11](controls how often the bond flipping move is triggered in OrganL). This is essential to get a minimal number of faces at the neck region (as necessary for constriction). This stable geometry is used as a starting point for all the other stomatocyte simulations (control, R_9 and K_9).

To scan the energy profile as a function of ν_0, we gradually increase V back to the initial value (corresponding to the sphere). Subsequently, each V value was sampled until the standard deviation in energy was minimized, ensuring convergence (at least 2×10^6 MC steps).

3.8 Analysis

- Energies are computed by averaging 1000 snapshots, corresponding to the 10^6 Monte Carlo steps, following an initial equilibration phase of at least 2×10^6 MC steps from the starting geometry.
- A *mesh averaging* procedure is applied purely for visualization purposes using the Iterative Closest Point (ICP) algorithm [19] over the last 200 output files. The face properties are averaged and mapped onto this ICP-generated mesh to produce the representative images included in the manuscript.
- To preserve spatial correlations, a *bin-averaging* approach is employed on the last 200 outputs (spanning 200 k MC steps). In this method, Property I from each snapshot is binned according to the values of Property II, and the binned values are subsequently averaged across all snapshots. This procedure ensures the retention of correlations between Property I and Property II, facilitating the analysis of effects such as mean and Gaussian curvature variations or lipid demixing in the presence of peptides, as discussed in the manuscript.

3.9 Results

Fig. S2 presents the complete energy decomposition, provided as supplementary information to complement the main plot in the primary manuscript (Figure 6).

Further, bin-averaged heatmap of the sorting mechanism on unfrozen meshes is plotted in Fig. S3. Compared with the frozen case in the manuscript (Fig. 7), it may be observed that this is very robust but not as pronounced, owing to the inclusion of mesh fluctuation.

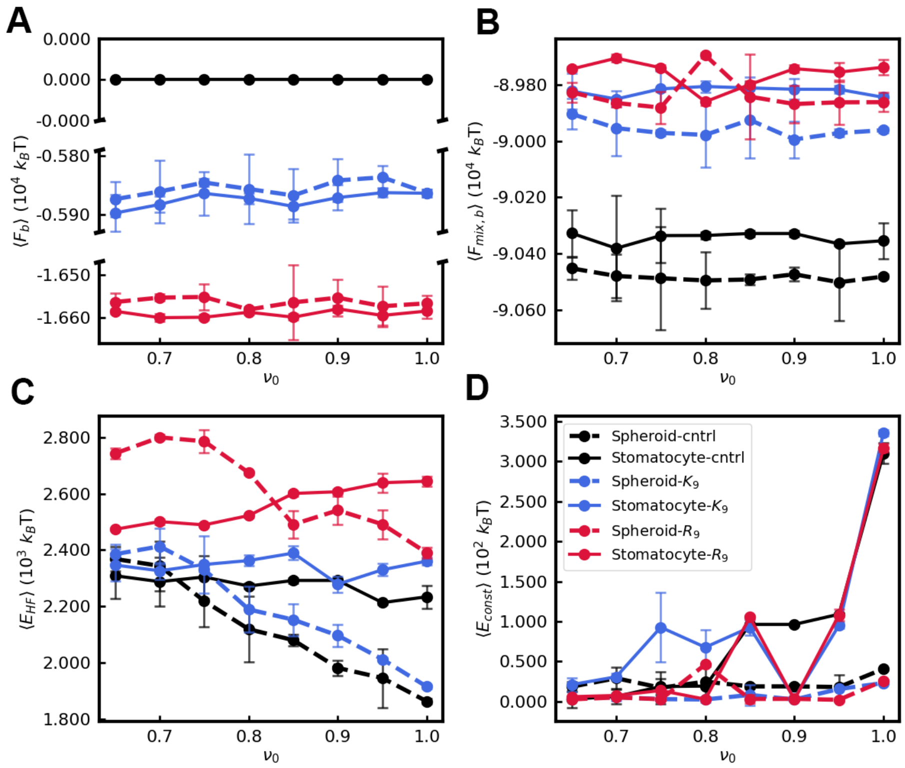

Figure S2: **Energy Decomposition Plot** Energy profiles of spheroid and stomatocyte morphologies in DOPE:DOPC:DOPS vesicles are presented under three conditions: without peptides (cntrl), with K_9, and in the presence of R_9. Error bars represent the SEM with 95% CI. (a) Binding Energy; (b) Mixing Entropy (c) Helfrich Energy and (d) Constraint Energy contributions respectively.

References

[1] Ricky Nencini, Carmelo Tempra, Denys Biriukov, Jakub Polák, Daniel Ondo, Jan Heyda, Samuli Ollila, Matti Javanainen, and Hector Martinez-Seara, *Biophys. J.*, **2022**, *121*, 157a.

[2] Christoph Allolio and Daniel Harries, *ACS Nano*, **2021**, *15*(8), 12880–12887.

[3] Rüdiger Goetz and Reinhard Lipowsky, *J. Chem. Phys.*, **1998**, *108*(17), 7397–7409.

[4] Marcello Sega, Balázs Fábián, and Pál Jedlovszky, *J. Chem. Theory Comput.*, **2016**, *12*(9), 4509–4515.

[5] Sukanya Konar, Hina Arif, and Christoph Allolio, *Biophys. J.*, **2023**, *122*(21), 4274–4287.

[6] P. Schofield, James R. Henderson, and John Shipley Rowlinson, *Proc. R. Soc. A*, **1982**, *379*(1776), 231–246.

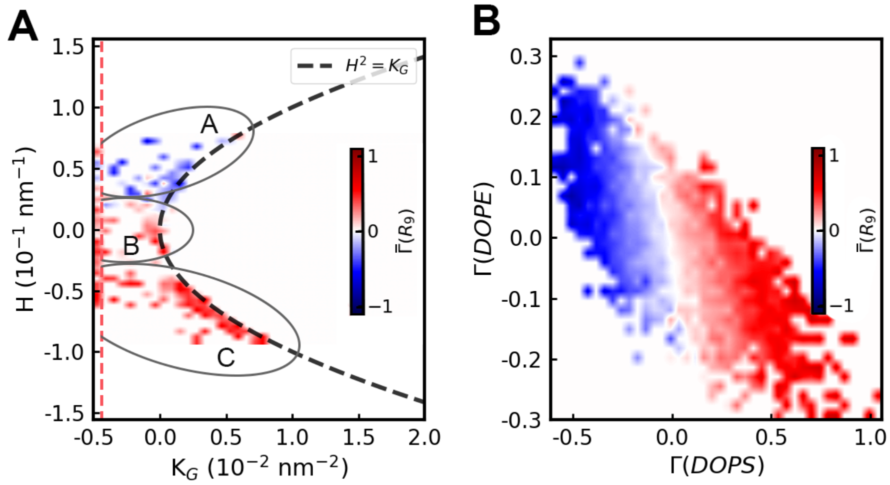

Figure S3: **Curvature Sorting on unfrozen mesh.** (a) Bin-averaged heatmap of excess R_9 coverage on stomatocyte at reduced volume (ν_0) of 0.80. Regions A, B, and C correspond to the exterior, neck, and invagination, respectively. (b) Heatmap showing the excess coverage of R_9 as a function of DOPE and DOPS composition. The dashed line delineates the excluded region by binning out all outliers to improve data clarity.

[7] Christoph Allolio, Amir Haluts, and Daniel Harries, *Chem. Phys.*, **2018**, *514*, 31 – 43.

[8] Igal Szleifer, Diego Kramer, Avinoam Ben-Shaul, William M. Gelbart, and S. A. Safran, *J. Chem. Phys.*, **1990**, *92*(11), 6800–6817.

[9] M. Hamm and M.M. Kozlov, *Eur. Phys. J. E*, **2000**, *3*(4), 323–335.

[10] S. A. Safran, P. A. Pincus, David Andelman, and F. C. MacKintosh, *Phys. Rev. A*, **1991**, *43*, 1071–1078.

[11] Christoph Allolio, Balázs Fábián, and Mark Dostalík, *Biophys. J.*, **2024**, *123*(12), 1553–1562.

[12] W. Helfrich, *Z. Naturforsch. C*, **1973**, *28*, 693–793.

[13] P.B. Canham, *J. Theor. Biol.*, **1970**, *26*(1), 61–81.

[14] Aaron D. Robison, Simou Sun, Matthew F. Poyton, Gregory A. Johnson, Jean-Philippe Pellois, Pavel Jungwirth, Mario Vazdar, and Paul S. Cremer, *J. Phys. Chem. B*, **2016**, *120*(35), 9287–9296.

[15] D. Andelman, M. M. Kozlov, and W. Helfrich, *Europhys. Lett.*, **1994**, *25*(3), 231.

[16] George Khelashvili, Daniel Harries, and Harel Weinstein, *Biophys. J.*, **2009**, *97*(6), 1626 – 1635.

[17] Mingyang Hu, John J Briguglio, and Markus Deserno, *Biophys. J.*, **2012**, *102*(6).

[18] Siegel D P and Kozlov M M, mar , **2004**, *87*(1), 366–374.

[19] K. S. Arun, T. S. Huang, and S. D. Blostein, *IEEE Trans. Pattern Anal. Mach. Intell*, **1987**, *PAMI-9*(5), 698–700.
